# The impact of thyroid function and thyroid autoimmunity on embryo quality in women with low functional ovarian reserve: a case-control study

**DOI:** 10.1186/s12958-015-0041-0

**Published:** 2015-05-15

**Authors:** Andrea Weghofer, Eric Himaya, Vitaly A. Kushnir, David H. Barad, Norbert Gleicher

**Affiliations:** Department of Obstetrics and Gynecology, Medical University Vienna, Waehringer Guertel 18-20, 1090 Vienna, Austria; The Center for Human Reproduction, 21E 69th street, New York, NY 10021 USA; Department of Obstetrics and Gynecology, Hospitalier de l’Université de Montréal, 2065, Rue Alexandre-de Sève, Montréal, Canada; The Foundation for Reproductive Medicine, 69th street, New York, NY 10021 USA

**Keywords:** Diminished Ovarian Reserve (DOR), Embryo quality, Euthyroid, *In Vitro* Fertilization (IVF), Low Functional Ovarian Reserve (LFOR), Thyroid autoimmunity, Thyroid Stimulating Hormone (TSH)

## Abstract

**Background:**

Women with hyper-and hypothyroidism are at increased risk for infertility and adverse pregnancy outcomes. Whether in women considered euthyroid thyroid function (TSH values) and thyroid autoimmunity (thyroid antibodies) influence *in vitro* fertilization (IVF) cycle outcome has, however, remained controversial. Any such effect should be easily visible in women with low functional ovarian reserve (LFOR) and thus small oocyte and embryo numbers.

**Methods:**

We evaluated the relationship between TSH levels and embryo quality in euthyroid women with LFOR undergoing IVF. Mean age for the study population was 39.9 ± 4.6 years. Embryo quality was assessed in 431 embryos from 98 first IVF cycles according to TSH levels (with cut-off 2.5μIU/mL), and to presence versus absence of thyroid autoantibodies.

**Results:**

Mean Anti Mullerian hormone (AMH) was 0.8 ± 0.8 ng/mL and mean TSH was 1.8 ± 0.9 μIU/mL. Comparable embryo quality was observed in women with TSH ≤ and >2.5μIU/mL. TPO antibodies significantly affected embryo quality in women with low-normal TSH levels (*P* = 0.045). In women with high-normal TSH levels, increasing TSH had a negative impact on embryo quality (*P* = 0.027). A trend towards impaired embryo quality with TPO antibodies was also observed in these patients (*p* = 0.057).

**Conclusions:**

TPO antibodies affect embryo quality in euthyroid women with low-normal TSH ≤2.5 μIU/mL. In women with high-normal TSH levels, increasing TSH levels, and possibly TPO antibodies, appear to impair embryo quality. These results suggest that the negative impact of thyroid autoimmunity becomes apparent, once thyroid hormone function is optimized.

## Background

Thyroid dysfunction is the most common endocrine disorder in women of reproductive years [1–3]. Hypothyroidism may cause menstrual irregularities and is believed to increase miscarriage rates [4–6].

Thyroid function and female reproduction are closely related. This relationship appears especially pronounced during pregnancy and in women who undergo fertility treatment: Once estrogen serum concentrations rise above physiological thresholds, thyroid-binding globulin levels increase. Thyroid-binding globulin binds thyroxin and leaves less free thyroid hormone available. Then thyroid-stimulating hormone (TSH) rises to ensure sufficient thyroid hormone supply [7, 8].

These observations led to the suspicion that thyroid function may influence *in vitro* fertilization (IVF) cycle outcomes. Whether clinical or subclinical hypothyroidism and/or thyroid autoimmunity affect female fertility and pregnancy potential in spontaneous and/or IVF cycles has remained subject of substantial disagreement [9–11].

Busnelli *et al.* recently demonstrated comparable implantation and live birth rates in IVF patients with clinical and subclinical hypothyroidism under levothyroxin treatment, and in presence and absence of thyroid autoimmunity [12]. Similarly, Chai *et al.* described comparable IVF pregnancy rates in women with normal TSH levels with thyroid autoimmunity and/or subclinical hypothyroidism [13].

Scoccia *et al.*, in contrast, reported significantly lower implantation and pregnancy rates in women with supplemented hypothyroidism undergoing assisted reproduction when compared to controls [8]. Monteleone *et al.* are in accordance with these findings. They reported lower fertilization and pregnancy rates in patients with thyroid autoimmunity when compared to controls [14].

Whether thyroid function and/or thyroid autoimmunity affect embryo quality has not yet been investigated. If thyroid function and/or thyroid autoimmunity affect embryo quality, such an effect would be especially apparent in women with low functional ovarian reserve (LFOR), who produce limited oocyte numbers during IVF.

To investigate the effects of thyroid function and thyroid autoimmunity on embryo quality in IVF patients with low functional ovarian reserve, the present study was initiated.

## Methods

### Patients

This study investigated 431 embryos in 98 infertility patients, who all underwent their first IVF cycles between January 2011 and February 2013 at the Center for Human Reproduction (CHR) in New York City.

Routine pre-IVF evaluation at the center includes ovarian function determinations by baseline follicle stimulating hormone (FSH) on cycle days 2/3 and random anti-Müllerian hormone (AMH), TSH and thyroid autoantibody assessments for thyroid peroxidase (TPO), anti-thyroglobulin (TG) and thyroid receptor antibodies (TR), and prolactin levels. All hormone assessments were made by routine commercial assays.

Only women with normal thyroid function (*i.e.*, TSH levels 0.45–4.5 μIU/mL) and normal serum prolactin were eligible for enrollment. Moreover, patients had to suffer from low functional ovarian reserve. A diagnosis of LFOR was established if women presented with abnormally elevated age-specific baseline FSH and/or abnormally low age-specific AMH. Normal age-specific hormone levels were defined as previously reported [15, 16]. Thyroid antibody status was considered positive in the presence of one or more thyroid antibodies.

### Laboratory assays

FSH, TSH and prolactin levels as well as TPO and TR antibodies were assessed by electrochemiluminescence immunoassay (ECLIA). AMH levels were measured by electrochemiluminescence (ECL). An immunochemiluminometric assay (ICMA) was utilized for anti-thyroglobulin and anti-thyrotropin receptor antibody measurements.

### IVF procedure

Since all here investigated patients suffered from LFOR, their ovarian stimulation was uniform, and included microdose GnRH agonist treatment (leuprolide acetate, Lupron®, Abbott Laboratories, North Chicago, Illinois) in 88.8 % of cycles. GnRH antagonist was applied in 11.2 % of cycles, usually only after patients had broken through with premature ovulation in a prior agonist cycle.

Ovarian stimulation routinely involved, as previously reported, FSH preponderance (300–450 I.U.) and a smaller dosage of daily human menopausal gonadotropin (hMG, 150 I.U.), with gonadotropin manufacturers depending on patient preference and insurance coverage [17]. When at least one follicle at 18–20 mm diameter was present, ovulation was induced with 10,000 I.U. of urinary human chorionic gonadotrophin (u-hCG).

After oocyte retrieval, oocytes are routinely incubated for at least 2 hours in an organ dish containing human tubal fluid (HTF) media (LifeGlobal, LLC, Gilford, CT) supplemented with 10 % human serum albumin (HSA, LifeGlobal, LLC, Gilford, CT). Then oocytes are stripped of cumulus cells with 80 IU/ml hyaluronydase (LifeGlobal, LLC, Gilford, CT) and by mechanically denudation.

Intracytoplasmic sperm injection (ICSI) is only performed on mature oocytes. Oocytes are placed into an ICSI dish containing 25 ul drops of HTF with HEPES, with 10 % of HSA added under mineral oil (LifeGlobal, LLC, Gilford, CT). Each drop is labeled. Following ICSI, oocytes are cultured individually under mineral oil in labeled drops (100 ul) of blastocyst media (LifeGlobal, LLC, Gilford, CT), supplemented with 10 % HSA. Dishes are prepared on the day prior to oocyte retrieval.

Quality of mature oocytes is documented by photography (INFINITY software, Lumenera Corp, Ottawa, Contario, Canada) prior to sperm injection and assessed during ICSI by the embryologist. Each oocyte is placed into a single-numbered drop in an ICSI dish, allowing for longitudinal assessments of subsequent fertilization and embryo development.

Microscopic evaluation of fertilization (or lack thereof) was performed approximately 18 h after ICSI. Zygotes were then incubated uninterrupted until day-3. On day-3 each embryo was individually evaluated by assessing blastomere numbers, embryo shape and fragmentation.

### Assessment of embryo quality

Embryo quality was assessed in a total of 431 embryos. Embryos were considered of good quality if they reached 4-cell stage on day 2 after fertilization and 8-cell stage with Grades 4 or 5 on day 3. They were considered of poor quality if they arrested, degenerated or only reached Grade 2; and they were considered fair if they fell in-between the first two groups [18]. Embryos of good quality were coded as 1, those of fair quality were coded as 2 and poor quality embryos were coded as 3. Due to limited embryo numbers in our center’s highly adversely selected patient population, embryo transfer is usually performed on day-3.

### Statistics

Thyroid function was measured by thyroid-stimulating hormone (TSH) levels. Thyroid autoimmunity was measured by the presence of at least one positive thyroid antibody test. Normal thyroid function was divided into low-normal and high-normal TSH levels with a cut-off of 2.5 μIU. This cutoff was previously reported by Busnelli *et al.* to distinguish between low-normal and high-normal TSH [9]. TSH levels were analyzed as continuous and as categorical variables. Embryo quality was measured as either poor, fair or good. Female age and oocyte numbers were determined as potential confounding factors.

A cumulative logit model was used while embryos were clustered by patient. All models passed the Pearson’s chi-squared goodness of fit test with non-significant p-values. Univariate analysis determined significant predictors which were then used in multivariate analysis. Pearson’s correlations with p-values are shown in Table 2.

Subgroup analyses were performed for the low-normal and the high-normal TSH groups Univariate analysis determined significant predictors which were then used in multivariate analysis (Table 3).

A 20 % difference in embryo quality between women with TSH ≤ 2.5 μIU/mL and those with TSH > 2.5 μIU/mL was estimated to be clinically significant. A power analysis was performed to calculate the number of embryos required per group to detect such a difference. To reach sufficient statistical power, 93 embryos per TSH group were required. The here presented study, therefore, clearly was of adequate size to detect such a 20 % difference in embryo quality.

Pregnancy and live birth rates were also assessed and reported, but not included in regression analyses, as case numbers were too small to draw valid conclusions.

Analyses were conducted using SPSS version 22. Covariates were considered statistically significant at *P*-values <0.05.

### Institutional review board

Here presented data only involved retrospective review of medical records and data from a de-identified research database. Patients at our center sign an informed consent at initial consultation, which allows for such reviews if the patient’s medical record remains confidential and her identity protected. These conditions were met in this case, allowing for expedited approval by the Institutional Review Board of The Center for Human Reproduction, NY, New York.

## Results

Patient and cycle characteristics are presented in Table 1. Mean age of all investigated patients was 39.6 ± 4.6 years, mean FSH was 11.4 ± 6.6 IU/mL, mean AMH 0.8 ± 0.8 ng/mL and mean TSH 1.8 ± 0.9 μIU/mL.

**Table 1:** Patient and IVF cycle characteristics of 98 euthyroid women suffering from diminished ovarian function and their 431 embryos according to TSH levels

	All	TSH ≤2.5μIU/mL	TSH >2.5μIU/mL
	(*n* = 98/431)	(*n* = 77/330)	(*n* = 21/101)
Female age (years)	39.6 ± 4.6	39.8 ± 4.5	38.8 ± 5.1
Baseline FSH (IU/mL)	11.4 ± 6.6	11.6 ± 6.9	10.5 ± 5.2
AMH (ng/mL)	0.8 ± 0.8	0.8 ± 0.8	0.8 ± 0.7
TSH (μIU/mL)	1.8 ± 0.9	1.5 ± 0.5	3.1 ± 0.4
Thyroid autoimmunity (%)	17.3 %	11.7 %*	38.1 %*
Thyroid peroxidase antibodies (%)	13.3 %	9.1 %**	28.6 %**
Thyroglubulin antibodies (%)	9.2 %	7.8 %	14.3 %
Thyroid receptor antibodies (%)	2.0 %	2.6 %	4.8 %
Total gonadotrophin dosage (IU)	6593.6 ± 2579.7	6613.3 ± 2599.2	6521.4 ± 2568.3
Number of oocytes retrieved	6.7 ± 4.4	6.7 ± 4.5	7.1 ± 3.7
Number of embryos transferred	2.6 ± 1.0	2.5 ± 1.0	2.7 ± 1.1
Good quality embryos (n, %)	203 (47.1 %)	152 (46.1 %)	51 (50.5 %)
Fair quality embryos (n, %)	183 (42.5 %)	141 (42.7 %)	42 (41.6 %)
Poor quality embryos (n, %)	45 (10.4 %)	37 (11.2 %)	8 (7.9 %)
Clinical pregnancy rate (%)	20.7 %	22.2 %	15.0 %
Live birth rate (%)	13.0 %	13.9 %	5.0 %

Values are presented as means ± SD

More than one thyroid antibody may be present in women with thyroid autoimmunity. Thus, percentages of the various thyroid antibodies may not add up to 100 %. Baseline and cycle characteristics are not statistically different between groups, if not indicated

*P = 0.015,**P = 0.003

Seventy-seven women (77.8 %) presented with TSH levels ≤2.5 μIU/mL and 21 (21.2 %) with TSH above 2.5 μIU/mL. Thyroid autoimmunity was present in 17 patients (17.3 %). Thyroid autoimmunity and TPO positive antibody status occurred more often in women with high normal TSH levels (*P* = 0.015 and *P* = 0.003, respectively). Mean thyroid peroxidase antibody values were 411.3 ± 769.5 IU/mL, mean thyroglobulin antibody levels were 146.4 ± 122.2 IU/mL and mean thyroid receptor antibody levels were 3.2 ± 0.8 IU/L. Mean prolactin levels were 12.7 ± 5.9 ng/mL.

After ovarian stimulation, a mean number of 6.7 ± 4.4 oocytes were retrieved. Among a total of 431 embryos, 203 (47.1 %) were classified as good, 183 (42.5 %) as fair and 45 (10.4 %) as poor quality embryos.

**Fig. 1:**
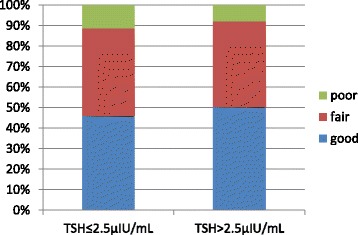
Embryo quality in references to TSH levels in 431 embryos from 98 first IVF cycles in 98 euthyroid women with low functional ovarian reserve

Embryo quality was not statistically different between TSH classifications of low-normal and high-normal TSH levels (Table 2, Fig. 1). Embryo quality correlated with oocyte numbers (*R* = −0.101;*P* = 0.036). A trend toward impaired embryo quality in the presence of thyroid autoimmunity was observed (*P* = 0.052). This trend was mainly attributable to the presence of TPO antibodies (*P* = 0.051). Multivariate regression, corrected for age and oocyte numbers, showed no significant correlation between embryo quality and TSH function and thyroid autoimmunity, respectively (Table 3).

**Table 2:** Pearson’s correlation of embryo quality with thyroid function and autoimmunity in 98 euthyroid women suffering from diminished ovarian function

	R	p
All TSH levels		
Age (years)	0.07096	0.1414
Number of oocytes	−0.10111	0.0359
TSH (μIU/mL)	0.02186	0.6509
TSH-cutoff (2.5μIU/mL)	−0.04928	0.3074
Thyroid autoimmunity	−0.09462	0.0515
TPO antibody status	0.09513	0.0508
TG antibody status	−0.01567	0.7491
Low-normal TSH levels (TSH ≤ 2.5μIU/mL)		
Age (years)	0.13800	0.0121
Number of oocytes	−0.11697	0.0337
TSH (μIU/mL)	0.07326	0.1844
Thyroid autoimmunity	−0.12046	0.0302
TPO antibody status	0.12856	0.0210
TG antibody status	−0.05218	0.3529
High-normal TSH levels (TSH > 2.5μIU/mL)		
Age (years)	−0.16302	0.1033
Number of oocytes	−0.06275	0.5331
TSH (μIU/mL)	0.24719	0.0127
Thyroid autoimmunity	0.00641	0.9495
TPO antibody status	0.07138	0.4803
TG antibody status	0.10443	0.3011

**Table 3:** Mulitvariate analysis of the impact of thyroid function and autoimmunity on embryo quality, controlled for age and oocyte numbers

	R^2^	P
All TSH levels		
TSH (μIU/mL)	0.0130	0.8159
Thyroid autoimmunity	0.0189	0.1509
TPO antibody status	0.0119	0.1141
TG antibody status	0.0142	0.8835
Low-normal TSH Levels (TSH ≤ 2.5μIU/mL)		
TSH (μIU/mL)	0.0301	0.2664
Thyroid autoimmunity	0.0330	0.2950
TPO antibody status	0.0404	0.0454
TG antibody status	0.0306	0.6836
High-normal TSH levels (TSH > 2.5μIU/mL)		
TSH (μIU/mL)	0.0782	0.0266
Thyroid autoimmunity	0.0548	0.1146
TPO antibody status	0.0663	0.0557
TG antibody status	0.0405	0.3053

Then potential impacts of thyroid function and thyroid autoimmunity on embryo quality were investigated among the low-normal and high-normal TSH subgroups. In women with low-normal TSH levels, female age (*P* = 0.012), oocyte numbers (*P* = 0.034), thyroid autoimmunity (*P* = 0.030) and TPO antibodies (*P* = 0.021) significantly affected embryo quality in univariate regression analysis. TPO antibody status significantly influenced embryo quality, when controlled for age and oocyte numbers (*P* = 0.045).

In women with high-normal TSH levels, increasing TSH levels were associated with impaired embryo quality (*P* = 0.013). This association remained significant in multiregression analysis. Additionally, a trend towards impaired embryo quality in women with TPO antibodies was observed (*P* = 0.056).

Among study participants, embryo transfer was performed in 93.9 % of women with a mean number of 2.6 ± 1.0 embryos transferred. The remaining patient cycles either demonstrated embryonic arrest or were eligible for embryo banking. The clinical pregnancy per transfer was 20.7 %, live birth rate was 13.0 %. Due to the small number of pregnancies, statistical analysis on the impact of baseline TSH levels on pregnancy potential was not feasible. However, a trend towards improved pregnancy potential in the presence of low-normal TSH levels (*i.e.* TSH ≤ 2.5 μIU/mL) was observed.

## Discussion

This study demonstrates comparable embryo quality in women with low-normal and high-normal TSH levels. Our results are in accordance with Reh *et al.*, who report comparable pregnancy, delivery and miscarriage rates among IVF patients with a TSH cut off of 2.5 μIU/mL [19]. These findings allow for different interpretations: One may assume that TSH levels within normal ranges have consistent effects on embryo quality [19]. Alternatively, one may speculate that the TSH cut off of 2.5 μIU/mL may be too low. Our data seem to support such an assumption: In women with high normal TSH levels, TSH increase was associated with poorer embryo quality. Such an assumption may also explain the conflicting results observed in the literature [9–12].

One may also assume that thyroid autoimmunity, rather than thyroid hormone concentrations, may impair IVF cycle outcomes [8]. Our findings appear to demonstrate such an effect: It appears that most of thyroid autoimmunity effect was due to positive TPO antibody status. Indeed, TPO antibodies significantly affected embryo quality in euthyroid women with low-normal TSH levels. The same trend was observed in women with high-normal TSH levels. These results deserve investigations to further elucidate the clinical significance of our findings.

That infertile women demonstrate a higher prevalence of autoimmunity than fertile controls [20, 21] further supports a causal relationship between thyroid autoimmune disease and impaired IVF cycle outcomes. Maternal tolerance needs to be established to facilitate embryonic implantation and autoimmunity appears to hinder this process [22]. It is tempting to speculate that embryo quality *and* implantation may be impaired in women with thyroid autoimmunity.

Higher miscarriage rates have also been reported in euthyroid women with signs of thyroid autoimmunity [14]. An effect that aligns well with impaired embryo quality we observed in the presence of TPO antibodies.

A recent meta-analysis by Velkeniers *et al.* concluded that thyroxine treatment in women with subclinical hypothyroidism and/or thyroid autoimmunity improves fertilization, implantation and live birth rate in women undergoing assisted reproduction. Additionally, significant decreases in miscarriage risk were reported. Women with thyroid autoimmunity in association with IVF also appear at increased risk of developing (sub-) clinical hypothyroidism [21]. These results are in accordance with our findings. Women with TSH > 2.5 μIU/mL levels were more likely to demonstrate thyroid autoimmunity (*P* = 0.015) and TPO antibodies (*P* = 0.003) than women in the lower TSH group. We also observed decreasing embryo quality with increasing TSH levels in patients with high-normal TSH levels.

## Conclusion

Our data demonstrate comparable embryo quality in LFOR women with low-normal and high-normal TSH levels. In women with low-normal TSH ≤2.5 μIU/mL, TPO antibodies negatively affect embryo quality. In women with high-normal TSH levels, increasing TSH levels, and possibly TPO antibodies, appear to impair embryo quality. These results suggest that the negative impact of thyroid autoimmunity becomes apparent, once thyroid hormone function is optimized.

## Abbreviations

AMH: Anti mullerian hormone
DOR: Diminished ovarian reserve
ECL: Electrochemiluminescence immunoassay
ECL: Electrochemiluminescence
FSH: Follicle stimulating hormone
ICMA: Immunochemiluminometric assay
IVF: *In vitro* fertilization
LFOR: Low functional ovarian reserve
TG: Thyroglobulin
TPO: Thyroid peroxidase
TR: Thyroid receptor
